# From bedside to bench: lung ultrasound for the assessment of pulmonary edema in animal models

**DOI:** 10.1007/s00441-020-03172-2

**Published:** 2020-02-03

**Authors:** Jana Grune, Niklas Beyhoff, Niklas Hegemann, Jonathan H. Lauryn, Wolfgang M. Kuebler

**Affiliations:** 1 Institute of Physiology, Charité-Universitätsmedizin Berlin, Charitéplatz 1, 10117 Berlin, Germany; 2 German Centre for Cardiovascular Research (DZHK), partner site Berlin, 10117 Berlin, Germany; 3 Institute of Pharmacology, Center for Cardiovascular Research, Charité-Universitätsmedizin Berlin, Hessische St 3–4, 10115 Berlin, Germany; 4 Department of Internal Medicine and Cardiology, Charité-Universitätsmedizin Berlin, Campus Virchow-Klinikum, 13353 Berlin, Germany; 5 The Keenan Research Centre for Biomedical Science at St. Michael’s, Toronto, Canada; 6 Departments of Surgery and Physiology, University of Toronto, Toronto, Canada

**Keywords:** Animal models, Lung ultrasound, Pulmonary edema, Diagnostics

## Abstract

Traditionally, the lung has been excluded from the ultrasound organ repertoire and, hence, the application of lung ultrasound (LUS) was largely limited to a few enthusiastic clinicians. Yet, in the last decades, the recognition of the previously untapped diagnostic potential of LUS in intensive care medicine has fueled its widespread use as a rapid, non-invasive and radiation-free bedside approach with excellent diagnostic accuracy for many of the most common causes of acute respiratory failure, e.g., cardiogenic pulmonary edema, pneumonia, pleural effusion and pneumothorax. Its increased clinical use has also incited attention for the potential usefulness of LUS in preclinical studies with small animal models mimicking lung congestion and pulmonary edema formation. Application of LUS to small animal models of pulmonary edema may save time, is cost-effective, and may reduce the number of experimental animals due to the possibility of serial evaluations in the same animal as compared with traditional end-point measurements. This review provides an overview of the emerging field of LUS with a specific focus on its application in animal models and highlights future perspectives for LUS in preclinical research.

## Background

Over the past decade, ultrasound and particularly lung ultrasound (LUS) has rapidly gained increasing importance as a monitoring and diagnostic tool in the intensive care unit (ICU) (Lichtenstein et al. 2014). LUS has been technically feasible for more than 30 years, yet due to its inability to provide accurate reflections of the organ’s anatomy, it was only applied by a few curious enthusiasts while most hospitals routinely excluded the lung from the ultrasound organ repertoire (Malbrain et al. 2017). Yet, for several lung disorders typically associated with intensive care settings such as pulmonary edema, pneumonia, or pleural effusions, LUS has recently proven its superiority over other diagnostic invasive and non-invasive imaging techniques such as chest radiography or physical examinations like auscultation. Specifically, LUS provides a higher diagnostic value, is more cost-effective and especially easy to perform directly at the patient’s bedside (Hendrikse et al. 2007; Lichtenstein and Mezière 2008; Durant and Nagdev 2010; Abdalla et al. 2016; Brogi et al. 2017). Consequently, the application of LUS as a diagnostic discipline in the ICU has rapidly increased and disseminated (Expert Round Table on Echocardiography in ICU 2014) due to (a) convincing results of clinical trials, demonstrating a predictive value for LUS in the diagnosis of lung disease, (b) standardization of protocols for the execution of LUS in the daily routine and in distinct patient cohorts and (c) the possibility of systematic LUS training focusing on the interpretation of complex and/or unusual LUS images for clinicians working in the ICU.

LUS’ recent implementation in clinical practice has sparked interest in a reverse translational approach for its analogous use in animal models with pulmonary edema formation, implementing and adopting the methodological experience from the clinics to preclinical applications. This review aims to draw attention to this newly emerging field of lung imaging in preclinical models of disease. As such, we discuss the potential of non-invasive LUS to diagnose pulmonary edema formation, acute respiratory distress syndrome (ARDS), pleural defects and effusion in standard of use animal models. To this end, we also provide a brief overview of different pathologies that can currently be diagnosed in the human setting by LUS. A special focus is given to the identification of pressure-mediated cardiogenic lung edema and permeability-type pulmonary edema as a result of alveolar-capillary barrier failure and the differentiation between the two.

### Basic principles of lung ultrasound

**Fig. 1:**
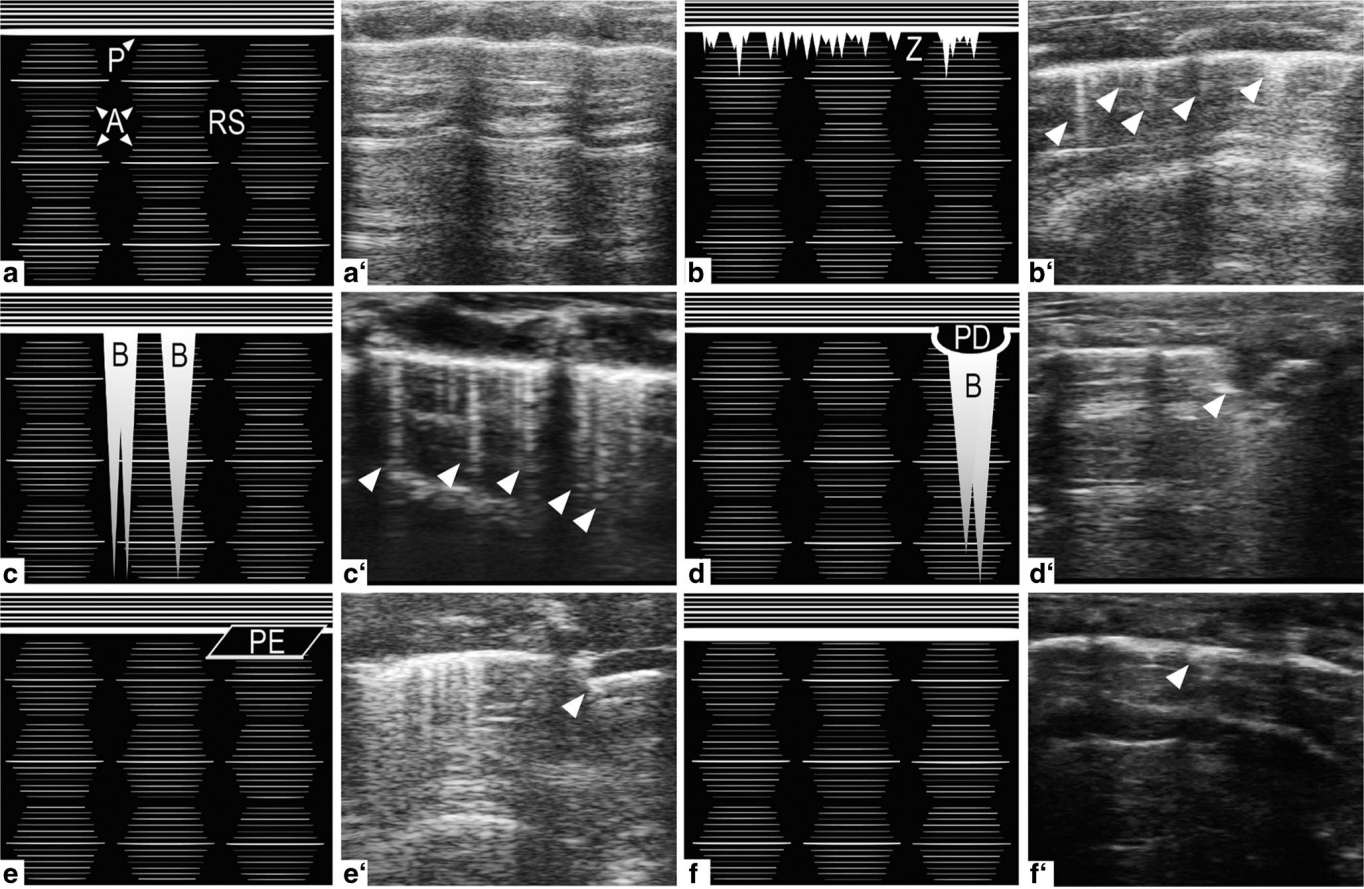
Physiologic and pathologic patterns in LUS B-Mode imaging. (a) Normally aerated lung with a distinct hyperechoic A-line (A) pattern subpleurally and regularly spaced rib shadows (RS). (b) Mostly aerated lung with small, hyperechoic, comet-tail artifacts arising downwards from the pleura (P), the Z-lines (Z). The association of Z-lines with disease states is presently unclear. (c) Partially aerated lung with long, A-line erasing, hyperechoic comet-tail artifacts called B-lines (B) arising downwards from the pleura indicating in this case the presence of alveolar-interstitial syndrome. (d) Pleural defect, resulting in a well-defined interruption of the pleural line. Occasional B-lines arising from the lower edge of the hypoechoic, defective area. (e) Hypoechoic pleural effusion (PE) between parietal (upper) and visceral (lower) pleura lines, which are otherwise indistinguishable by LUS imaging; commonly associated with conditions like heart failure (transudate) or pulmonary embolism (exudate). Also note the presence of Z- and B-lines on the left side of the LUS image. (f) Pleural thickening, indicating the presence of fibrotic or inflammatory lesions

The physical rationale of ultrasound imaging is based on the reflection of sound waves when crossing the border between two different media (Wachinger et al. 2008). Depending on their individual acoustic impedance, a measure for resistance to tissue oscillation, a defined portion of sound waves, will be reflected back towards the transducer and another will travel further into the object or tissue. The mentioned reflections, so-called echoes, will project an image seen on the ultrasound device (Hangiandreou 2003). In classic ultrasound imaging such as echocardiography, these echo signals can be used to generate a time-resolved accurate anatomical depiction of the organ of interest. In contrast, visualization in LUS is mainly limited to ultrasound artifacts. The co-appearance of soft tissues and air in the lung will reflect ultrasound waves almost completely and will therefore not generate an image but only unique ultrasound artifacts that are described in depth later. Fluids transmit ultrasound waves with only minimal reflection; therefore, they do not generate an echo and appear as black areas (Abu-Zidan et al. 2011). As a result, different compositions of healthy and diseased lungs generate different artifactual patterns in LUS that can be utilized for diagnostic purposes (Fig. 1) (Miller 2016). For example, Fig. 1(a) depicts normally aerated lungs, whereas Fig. 1(b–f) all present with distinct visual differences characteristic for specific pulmonary diseases. In the following paragraphs, we briefly introduce the physiological and pathological patterns detected in LUS imaging.

#### LUS in healthy lungs

**Fig. 2:**
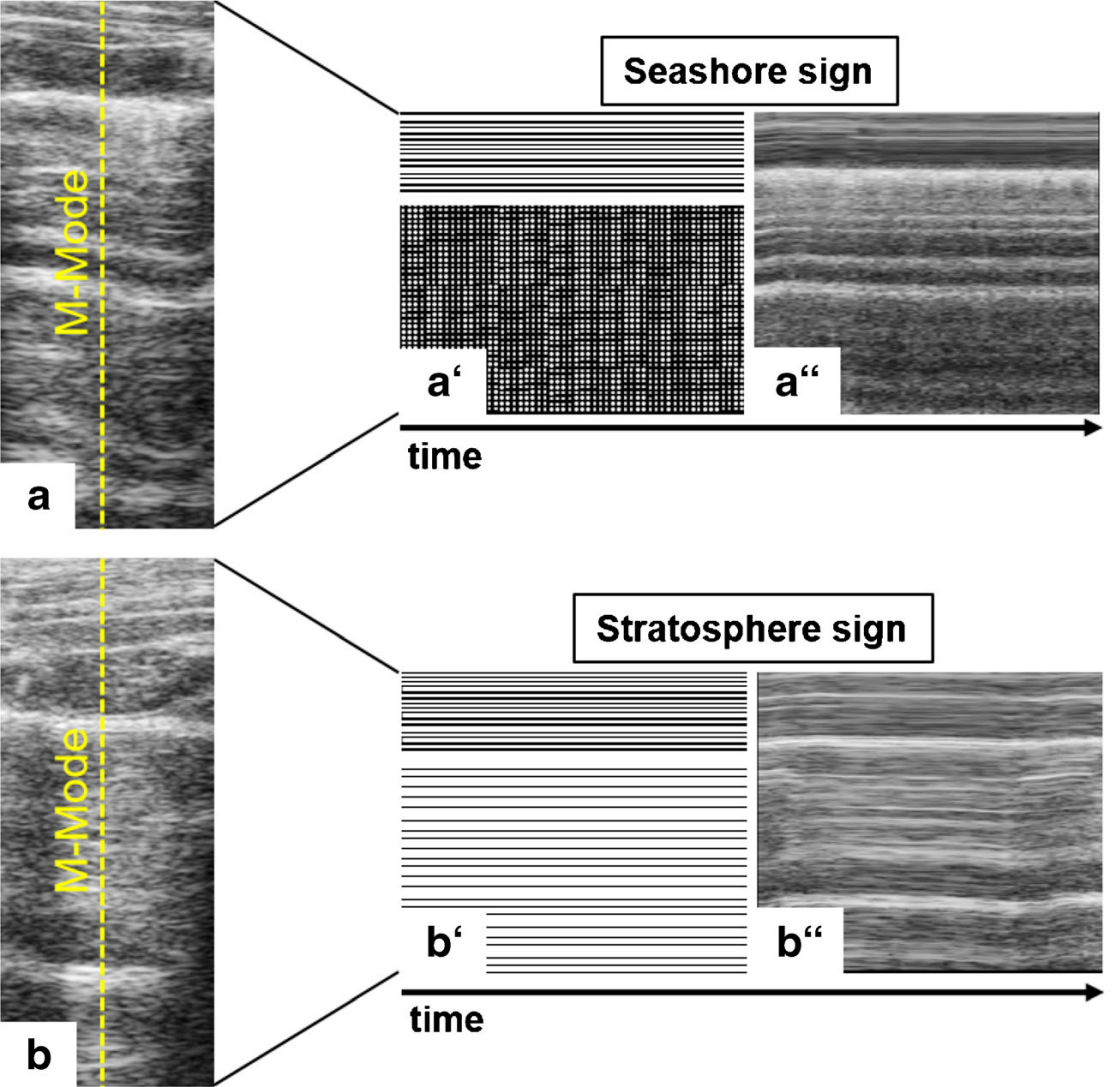
M-Mode imaging across supra- and subpleural spaces over time in LUS. (a) Seashore sign: LUS presents suprapleurally with continuous wave-like lines and a diffuse, sand-like pattern subpleurally, indicating physiologic movement of lung tissue during respiration. (b) Stratosphere sign: lung movement is absent, suggesting the occurrence of a pneumothorax

In echographic B-Mode imaging, normally aerated lungs (Fig. 1a) present with a distinct pattern showing a notable hyperechoic horizontal line representing the pleura as an important landmark in the upper third of the image. A phenomenon called lung sliding describes the back and forth movement of the pleural line in sync with respiration (Volpicelli et al. 2012). Structures seen above this line constitute subcutaneous tissue, intercostal muscles and ribs (Rippey and Gawthrope 2012). Below the pleural line, normal lung tissue is represented by regular spaced, white, horizontal hyperechoic reverberation artifacts and A-lines. A-lines are generated by ultrasound waves repeatedly bouncing back and forth between the pleura and transducer. As compared with the first reflection, subsequent reflections reach the transducer with a temporal delay and are erroneously interpreted and displayed as signals from deeper within the lung tissue. Consequently, the distance between the superimposed A-lines is always equidistant to or a multiple of the distance between the pleura and the transducer surface (Shojaee and Argento 2014; Saraogi 2015). Recurrent vertical hypoechoic spaces between A-line conglomerates are caused by ribs that do not allow for the ultrasound waves to penetrate further, generating a downward shadow in the ultrasound image (Villalba-Orero et al. 2017). Using the M-Mode across the supra- und subpleural space of a healthy lung generates the so-called seashore sign (Fig. 2a) (Stone 2008) with continuous wave-like lines in the suprapleural space. The subpleural space shows an undefined pattern reminiscent of sand on a shore. In fact, the “sandy” artifact results from lung sliding, i.e., the movement of the lung during respiration (Gargani 2011).

#### Pathological findings in LUS

A number of pulmonary pathologies result in various irregularities in these patterns. The following paragraphs will briefly summarize these disease-associated findings and their clinical correlates.

Z-lines (Fig. 1b) are small and short hyperechoic, comet-tail artifacts arising from the pleural line downwards. These artifacts are likely caused by resonance between the parietal pleura and endothoracic fibrous connective tissue (Lee 2016). Z-lines flicker and do not move along lung sliding. Cumulative evidence does not attribute clinical significance to Z-line detection during LUS imaging and indicates that the majority of healthy individuals present with Z-line patterns (that are eventually mistaken for B-lines) (Lee 2016; Francisco et al. 2016). Lichtenstein and colleagues similarly reported the occurrence of Z-lines in healthy individuals as well as patients suffering from a pneumothorax, also questioning the diagnostic value of Z-line findings (Lichtenstein et al. 2009). While Z-lines are considered by many a normal finding unrelated to pulmonary pathologies, others have proposed Z-lines to precede B-lines (next paragraph) and as such, to be already associated with pulmonary diseases such as, e.g., pneumothorax (Lichtenstein et al. 2009; Lee 2016; Villalba-Orero et al. 2017).

B-lines (Fig. 1c) on the other hand are unanimously considered a pathological finding (Dietrich et al. 2016; Picano and Pellikka 2016). B-lines present as long comet-tail artifacts that also arise from the pleural line, do not fade and erase A-lines on their way down on the screen. In addition, B-lines move jointly with lung sliding. This artifact is generated by thickening of interlobular septa due to edema or ground-glass lesions. In either case, the different acoustic impedance causes a reverberating reflection of the ultrasound beam. The short time between each reverberation results in the very tight spacing of B-lines, giving them a sunbeam-like appearance (Lichtenstein et al. 1997). Accordingly, the presence of B-lines in LUS can indicate a number of different pulmonary pathologies that feature interlobular septal thickening or pulmonary lesion formation, such as alveolar-interstitial syndrome or ARDS (Yang et al. 1992; Lichtenstein et al. 2009; Wongwaisayawan et al. 2016). As such, B-lines can reflect edematous changes in the lung even prior to the onset of alveolar flooding, thus affirming not only the diagnostic but also prognostic value of LUS (Lichtenstein et al. 1997).

Further commonly diagnosed pathologies in clinical LUS are pleural irregularities (Fig. 1d–f) as pleural effusion, thickening, or other defects generate fundamentally different diagnostic findings (Sehgal et al. 2016). In case of effusion, a black, swollen-looking pouch forms within the pleural cavity between the parietal and visceral pleura, which are otherwise indistinguishable by LUS under physiological conditions (Lichtenstein 2017). This pouch constitutes a pathological accumulation of fluid transudate or exudate in the pleural cavity (Karkhanis and Joshi 2012). As echogenicity increases with increasing density of a material, fluid will appear black due to the transmission of ultrasound waves with only minimal reflection (Abu-Zidan et al. 2011). Among the most common causes for pleural effusions are tumors, congestive heart failure, pneumonia and tuberculosis (Porcel et al. 2014). Pleural thickening, on the other hand, appears to be either focal, representing fibrotic or inflammatory lesions, or diffuse in combination with pleural effusions. Thickening of the pleura caused by benign or malignant tumors is relatively rare and presents with different echogenic characteristics depending on the structural composition of the tumor mass (Rumende 2012).

In contrast to the seashore sign found in M-Mode imaging of normally aerated lungs, the stratosphere sign (Fig. 2b) describes the state of immobile lung tissue in the subpleural space (Stone 2008). The M-Mode image therefore lacks the “sand” and presents continuous hyperechoic lines both supra- and subpleurally. The stratosphere sign is typically associated with a diagnosis of pneumothorax (Stone 2008).

Pulmonary consolidations describe the filling of small airways or distal air spaces with fluid, puss, blood, cells, or other material. In some cases, primarily seen with lobar pneumonia, these consolidations present in LUS by a hypoechoic, liver-like appearance called lung hepatization (Gehmacher et al. 1995). The so-called bronchograms occasionally accompany consolidations and may appear in LUS either as hyperechoic (trapped air) or hypoechoic (trapped fluid) bronchiolar structures (Ho et al. 2015). Bronchograms are most commonly caused by pneumonia or pulmonary edema as a consequence of heart failure or ARDS (Durant and Nagdev 2010). Notably, in case of pulmonary consolidations, B-lines may not solely arise from the pleural line but also from subpleural consolidated areas, similar to those seen with pleural defects (Stadler et al. 2017).

The above described disease-associated findings in LUS often present in various combinations depending on the underlying condition, which expands the range of possible diagnostic patterns seen in LUS.

### Clinical application of lung ultrasound

At first sight, LUS may appear complex and unintuitive, yet this notion should be more than outweighed by its demonstrated clinical usefulness. In general, LUS generates highly standardized, reproducible patterns, which explain the high interobserver agreements between operators. A study of Lichtenstein et al. investigated interobserver variabilities in 288 lung regions of an ARDS-patient cohort by using the κ reliability test. κ values for assessing a normal lung ultrasonography pattern, alveolar-interstitial syndrome, alveolar consolidation, and pleural effusion were between 0.69 and 0.77 indicating medium to high agreement rates between two independent observers (Lichtenstein et al. 2004). For clinicians, beginning their LUS training on critically ill patients, the detection of pleural effusions and lung consolidations in corresponding lung regions is the easiest part and basic skills are generally acquired over a short period of time (Doelken and Strange 2003). Once the process has been learned, a step-by-step use enables implementation of LUS into clinical routine. This implementation is not limited to the ICU (Mojoli et al. 2018) but extends to other scenarios and disciplines such as transthoracic echocardiography in clinical cardiology (Picano et al. 2018). By now, a series of studies show that LUS is not only able to diagnose several lung diseases but moreover, provides a useful tool in their differentiation against each other, thus contributing to adequate therapy (Bitschnau and Mathis 1999; Maury et al. 2001; Rowan et al. 2002; Reissig and Kroegel 2003; Mayo et al. 2004; Soldati et al. 2006; Volpicelli et al. 2006; Fagenholz et al. 2007). In the following paragraphs, we will describe two examples of clinical applications of LUS to highlight its potential as a diagnostic tool in the ICU.

#### The BLUE protocol

A primary objective of clinicians in the ICU is to save time during the process of diagnosis and treatment decision-making. Especially patients with acute respiratory failure, a severe life-threatening situation relies on rapid diagnosis, highlighting the clinical need for sophisticated techniques to inform disease management. However, emergency patients frequently present in conditions far from ideal for immediate diagnosis, hampering the clinical outcome (Wasserman 1982; Aronchick et al. 1985; Ray et al. 2006). LUS ideally matches the clinical need for rapid bedside diagnosis, since it is broadly available, cost-effective, non-invasive and takes only a couple of minutes (Bouhemad et al. 2007). The groundbreaking study of Daniel Lichtenstein and Gilbert Mezière, which became known as the BLUE protocol (*Bedside Lung Ultrasound in Emergency*) compared LUS results on initial presentation of 260 dyspneic patients with the final diagnosis by the ICU team in order to assess the potential of LUS to diagnose acute respiratory failure (Lichtenstein and Mezière 2008). The authors used only three LUS signs with dual answers for diagnosis: artifacts, lung sliding and pleural effusion and/or alveolar consolidation. The 3 criteria of LUS were used to diagnose and discriminate patient cohorts in a retrospective manner and to specify sensitivity (true positive rate) and specificity (true negative rate) of LUS (Table 1). For example, multiple anterior diffuse B-lines with lung sliding indicated pulmonary edema with a sensitivity of 97% and a specificity of 95%. Further, the authors were able to discriminate patient cohorts with COPD (chronic obstructive pulmonary disease) or asthma, pulmonary embolism, pneumothorax, or pneumonia with an overall correct diagnosis in 90.5% of cases when compared with conventional diagnostic tools (Lichtenstein and Mezière 2008).

**Table 1:** Accuracy of the BLUE protocol (adopted from Lichtenstein 2015, p)

Cause of dyspnea	Sensitivity (%)	Specificity (%)
Acute hemodynamic pulmonary edema	97	95
Exacerbated COPD or severe acute asthma	89	97
Pulmonary embolism	81	99
Pneumothorax	88	100
Pneumonia	89	94

#### The FALLS protocol

A main product of the BLUE protocol was the FALLS protocol (*Fluid Administration Limited by Lung Sonography study*), which aimed to address the unmet clinical need of diagnosing the underlying cause in a patient with unexplained acute shock syndrome in the ICU (Lichtenstein 2012, 2013). Specifically, septic shock is one of the most common and serious complications in the ICU (Angus et al. 2001) and despite numerous consensus statements remains challenging to diagnose in the absence of a validated standard diagnostic test (Singer et al. 2016). According to the 2016 Consensus paper on sepsis and septic shock (Sepsis-3), the severity of organ dysfunction in patients with suspected infections is commonly assessed by scoring systems that quantify abnormalities of clinical findings, laboratory data, or therapeutic interventions. This approach, however, can result in considerable differences depending on the individual scoring system used and/or due to inconsistencies in reporting the patient’s clinical status (Singer et al. 2016). The FALLS protocol demonstrated that imaging artifacts of LUS can assist in the differential diagnosis of affected patients and facilitate treatment decision-making in terms of fluid administration (Lichtenstein 2013). The latter is of critical relevance, as on the one hand, early goal-directed therapy with aggressive fluid resuscitation in the first 6 h of diagnosis can reduce mortality and both hospital and ICU length of stay in patients with sepsis (Angus et al. 2001; Bouchard and Mehta 2010), yet fluid overload on the other hand, defined by a cutoff value of 10% of fluid accumulation, is equally associated with a worse outcome in septic shock (Malbrain et al. 2018).

Specifically, the FALLS protocol describes a workflow using ultrasound technology that assists in the diagnosis of septic shock by sequential elimination of other shock etiologies in a hierarchical order. First, obstructive shock is ruled out by a negative diagnosis of pericardial tamponade or pulmonary embolism using cardiac sonography. Second, if the so-called lung rockets (multiple B-lines) are absent in LUS, the diagnosis of cardiogenic shock should be discarded (Lichtenstein 2012). If the patient is diagnosed with an A-profile in LUS (normal sonographic lung surface), he or she is defined as a FALLS responder and will receive fluid therapy. An improvement of patient symptoms and unchanged A-lines is suggestive of hypovolemic shock conditions. Vice versa, a lack of improvements or even the transformation from horizontal A-lines to vertical B-lines points towards fluid overload and the development of ARDS in conjunction with severe sepsis (Lichtenstein 2012, 2013). A comparison between *BLUE* and *FALLS* protocol can be found in Table 2.

**Table 2:** Comparison of *BLUE* and *FALLS* protocol (Lichtenstein and Mezière 2008; Lichtenstein 2012, 2013)

*BLUE*		*FALLS*	
LUS sign	Diagnosis	LUS sign	Diagnosis
Lung sliding: present B-profile	Pulmonary edema	Emergency cardiac sonography: pericardial tamponade RV dilatation *BLUE* protocol: pneumothorax (A-profile)	Ruling out obstructive shock
Lung sliding: any A/B-profile	Pneumonia	*BLUE* protocol: pulmonary edema (B-profile)	Ruling out (left) cardiogenic shock
Lung sliding: abolished B-profile	Pneumonia	Correction of clinical signs of shock under fluid administration (A-profile)	Ruling out hypovolemic shock
Lung sliding: abolished A-profile	Pneumothorax	Fluid therapy not able to improve circulation—eventually generating a B-profile	Detecting distributive shock (septic shock usually)
Lung sliding: present A-profile Sequential venous analysis: thrombosed vein	Pulmonary embolism		
Lung sliding: present A-profile Sequential venous analysis: free vein	Pneumonia, COPD, or asthma		

*RV*, right ventricle

These examples demonstrate the clinical usefulness of LUS, in particular when compared with computed tomography (CT), which is cost- and time-intensive, resulting in delayed care implementation, requires supine positioning of patients and exposes them to irradiation (Brenner et al. 2001; Brenner and Hall 2007). Indeed, when compared with thoracic CT and bedside chest radiography, LUS is almost equivalent in detecting the main lung pathologic entities in patients with ARDS and can provide additional information in terms of superior focal resolution (Table 3) (Lichtenstein et al. 2004; Lichtenstein and Peyrouset 2006).

**Table 3:** Comparison of CT scan (CT), chest X-ray (CXR), lung ultrasound (LUS) and respiratory examination consisting of inspection, palpation, percussion and auscultation (RE) as techniques for the assessment of pulmonary status

Technique	Strengths	Weaknesses
CT scan	- Gold standard - Highest diagnostic value	- Irradiation - Bedside-systems very rarely available - High costs - Long acquisition and interpretation (hours) - Radiologist required
Chest X-ray	- Commonly used and widely accepted	- Irradiation - Limited access to bedside-compatible systems - Medium to high cost - Lower diagnostic sensitivity, specificity and accuracy than LUS and CT scan - Long acquisition and interpretation (hours) - Radiologist required
Lung ultrasound	- Bedside performance - Relatively low cost - Standard ultrasound machine commonly available with general practitioners and clinics - High diagnostic and prognostic value - Fast (minutes)	- Appropriate lung ultrasound training required - Deep tissue lesions might not be picked up
Respiratory examination	- Bedside performance - Required skills part of general medical training - Only stethoscope needed - Low cost - Fast (minutes)	- Limited diagnostic value - Often additional assessments required for detailed diagnosis - Difficult in unconscious/comatose patients

### Preclinical application of lung ultrasound for detection of lung edema

The successful application of LUS as a diagnostic tool in the clinics has recently sparked interest in its potential use for preclinical monitoring in a reversed translation approach. So far, the number of small animal studies with LUS is still limited but can be expected to grow exponentially based on previous clinical experience and the precedent of small animal echocardiography that underwent a similar reversed translation. Preclinical studies so far have focused predominantly on the use of LUS for the detection and discrimination of pulmonary edema. In the following paragraphs, we will very briefly recapitulate the pathophysiology of pulmonary edema and then summarize the present state-of-the-art for the use of LUS in its preclinical diagnosis and monitoring.

#### Pathophysiology of pulmonary edema

Pulmonary edema describes the pathological accumulation of extravascular lung water (EVLW) resulting from an imbalance between fluid filtration and resorption that exceeds the physiological fluid flux from the vasculature to the interstitial space (Ware and Matthay 2005). Under physiological conditions, filtrated fluid in the interstitial space is predominantly reabsorbed via blind ending lymphatic vessels, located in peribronchovascular, interlobular septa and in the subpleural space (Zarins et al. 1978; Pearse et al. 1993). Pulmonary edema ensues when fluid filtration exceeds the capacity of the lymphatic system to clear the filtrated fluid from the interstitial space (= cardiogenic edema, resulting from increased hydrostatic pressures) and/or when fluid transport across the alveolar endothelium and epithelium becomes dysregulated due to alveolar-capillary barrier failure and/or impaired epithelial fluid absorption (= permeability-type edema, resulting from infectious, inflammatory, or mechanical injury) (Ware and Matthay 2005). While pathophysiology and, thus, treatment differ essentially between these two types of pulmonary edema, the similarity of the clinical presentation frequently complicates appropriate diagnosis and differentiation. Hence, underlying etiologies have to be evaluated, stressing the importance of focusing on a patient’s history for appropriate therapy. This is particularly noteworthy as the presence of pulmonary edema in critically ill patients is associated with higher morbidity, prolonged ICU stays and requirement for mechanical ventilation. Pulmonary edema thus presents a significant burden on the health care system and, more importantly, typically signifies a worsening in the patient’s medical prognosis (Edoute et al. 2000; Sakka et al. 2002; Dasta et al. 2005).

#### LUS for diagnosis of clinical permeability-type edema

Chest roentgenograms and CT are widely used for the diagnosis of permeability-type edema and bilateral opacities on chest images, which constitute one of four criteria for the Berlin definition of ARDS (ARDS Definition Task Force et al. 2012). Yet, despite their widespread use, considerable limitations exist that pertain to the lack of EVLW quantification and its real-time assessment, radiation exposure, interobserver variability and last but not least safety concerns regarding patient transportation out of an ICU for CT scans (Pistolesi and Giuntini 1978; Sibbald et al. 1983; Halperin et al. 1985; Meade et al. 2000; Rubenfeld et al. 2005; Warren et al. 2018).

Over the past two decades, two new diagnostic options for the diagnosis of pulmonary edema have become available, namely invasive transpulmonary thermodilution (TPTD) and LUS. TPTD allows for an exact quantification of EVLW by measuring the transition time of a cold saline injection from the central venous catheter to a femoral artery catheter (Sakka et al. 2002; Katzenelson et al. 2004; Kirov et al. 2004; Rossi et al. 2006). While real-time data on EVLW can help to optimize fluid management in critically ill patients, the need for invasive catheterization may give rise to potential complications such as hemorrhage or arterial injury and thrombosis, which is frequently not feasible in an emergency setting. LUS, on the other hand, offers a less quantitative but often more feasible and fast choice for assessing acute pulmonary edema in emergency settings or at the bedside when roentgenograms or CTs are not available—i.e., in the majority of medical facilities around the globe. LUS allows for semi-quantitative assessment as the number of B-lines correlates with EVLW rendering LUS a potential alternative to chest roentgenograms or CT (Lichtenstein et al. 1997; Jambrik et al. 2004) as well as TPTD (Agricola et al. 2005). An additional advantage of LUS is the smaller interobserver variability compared with chest roentgenograms (Touw et al. 2018).

Different from other techniques, LUS provides real-time data on permeability-type edema, as demonstrated by its ability to detect immediate decreases in EVLW in patients undergoing hemodialysis (Noble et al. 2009; Trezzi et al. 2013). Detecting the volume load and the response to fluids, whether it will be in hemodialysis or ICU patients, is crucial in fluid therapy adjustments. Unfortunately, clinical signs like crackles at auscultation or pitting edema only have a low sensitivity for the semi-quantitative detection of volume overload. In contrast, the recent LUST study identified B-lines in LUS to be superior in detecting pulmonary congestion and to discriminate the amount of EVLW in dialysis patients at high cardiovascular risk compared with standardized lung auscultation (Torino et al. 2016). Moreover, the number of B-lines correlated with physical function, predicted cardiac events and mortality in hemodialysis patients (Zoccali et al. 2013; Enia et al. 2013). Accordingly, B-lines offer a fast and accessible way of determining the hydration status and subsequently the correction of fluid removal in dialysis and critically ill patients (Jiang et al. 2017). These findings highlight the potential role for LUS to guide fluid therapy and for early diagnosis of overhydration. As such, LUS can address a considerable unmet medical need, as volume overload poses an independent risk factor for death due to cardiovascular events in dialysis patients (Saad et al. 2018).

Not only is LUS a faster way of assessing EVLW but it has also been used successfully to differentiate between ARDS (as a prototypic form of permeability-type edema) and cardiogenic edema (Copetti et al. 2008). While cardiogenic edema presents as a uniform distribution of B-lines, LUS findings in ARDS patients show a heterogeneous distribution of B-lines. Furthermore, cardiogenic edema presents with normal lung sliding and homogenous pleural effusions, while ARDS-findings in LUS include pleural line abnormalities, lack of lung sliding, uneven tissue patterns such as “spared areas” and consolidations. In addition, consolidation-associated findings like air bronchograms and “lung pulses,” i.e., absent lung sliding with visible cardiac motion at the pleural line, can be observed (Copetti et al. 2008; Assaad et al. 2018). In contrast to other techniques, LUS provides real-time data on permeability-type edema, as demonstrated by its ability to detect immediate decreases in EVLW in patients undergoing hemodialysis (Noble et al. 2009; Trezzi et al. 2013). The sensitivity of LUS is highlighted by its ability to detect alveolar edema significantly earlier as compared with resulting ventilation/perfusion mismatches and consecutive changes in oxygenation defined by the ratio of partial pressure of arterial oxygen to the fraction of inspired oxygen (PaO_2_/FiO_2_) (Caltabeloti et al. 2014).

#### LUS in experimental permeability-type edema

Over the past years, LUS has thus emerged as a powerful technique, widely accessible and easy to master, which can provide clinically meaningful information towards the pulmonary status. This clinical success has, however, not been matched in preclinical research, as use and validation of LUS in animal models of permeability-type edema has been limited so far. State-of-the-art for the diagnosis of acute lung injury in rodents is a composite measure of histological evidence of tissue injury, impaired alveolar-capillary barrier function, inflammation and physiological dysfunction (Matute-Bello et al. 2011). As these parameters are largely assessed by end-point measurements at the time of animal sacrifice, LUS may offer significant advantages in terms of non-invasive longitudinal studies. The possibility to translate LUS to experimental animal studies was first documented by Jambrik and colleagues who applied LUS in minipigs in a model of oleic acid-induced acute lung injury (ALI), the preclinical equivalent to human ARDS. In this study, the number of B-lines assessed in vivo correlated significantly with lung wet-to-dry weight ratios as determined gravimetrically postmortem (Jambrik et al. 2010). LUS has also proven to be consistent with human studies in a porcine model of oleic acid-induced ALI regarding early prediction of ventilation/perfusion mismatches (Gargani et al. 2007). Taking it one step further to the level of rodent models, Ma and coworkers tested LUS in male Sprague-Dawley rats before and after a challenge with lipopolysaccharide (LPS) to induce ALI/ARDS (Ma et al. 2016). To this end, they scanned the dorsal wall in supine position bilaterally at 4 different locations using a score of maximal 10 points per scanning position for the number of B-Lines. Over the time course of the experiment, normal A-patterns in LUS were replaced by a dose-dependent increase in B-lines, with a later confirmation of ALI/ARDS-induction by PET/CT and in a supplementary study also by gravimetrically determined wet-to-dry lung weight ratios (Ma et al. 2015, 2016).

Despite these promising results from first pioneering studies, relevant limitations apply, as image acquisition and interpretation can be afflicted by, e.g., body position-dependent accumulation of edema fluid resulting in inhomogeneous speckled appearances or due to inexperienced sonographers. To overcome such limitations from visual analysis, Corradi and coworkers postulated that the amount of EVLW might be more reliably assessed using a computer-assisted gray-scale analysis (Corradi et al. 2013). To test this hypothesis, the authors reproduced edema formation in isolated, intubated and ventilated bovine lungs that were consecutively instilled intrabronchially with 0.45% NaCl solution and examined by CT and LUS. The chest wall was mimicked by a chloroprene water-filled bag, the complete surface was scanned in a video-based quantitative manner at a constant velocity and a 90° angle and Jambrik’s scoring system was applied for automated analysis of LUS recordings (Jambrik et al. 2010). Surprisingly, quantitative LUS computer-assisted gray-scale analysis outperformed CT analysis by mean attenuation in Hounsfield units in the detection of EVLW (Corradi et al. 2013). While further in vivo experiments are required to validate these findings in in vivo settings of actual permeability-type or cardiogenic lung edema (rather than intrabronchial instillation in isolated lungs), these results hold promise for video-based approaches and automated image analysis that may not be restricted to animal experiments but also eventually applicable to humans as an exemplary case of reverse-reverse translation.

#### LUS for diagnosis of clinical cardiogenic edema

Cardiogenic edema is caused by increased transvascular fluid filtration across an intact endothelial barrier resulting—in contrast to permeability-type pulmonary edema—in a protein-poor and cell-free edema fluid in the alveolar space (Fein et al. 1979). Cardiogenic edema develops as a result of elevated hydrostatic pressure in the pulmonary capillaries, a hemodynamic effect most commonly caused by acute or chronic left-sided heart failure. Heart failure (HF) is a leading cause of morbidity and mortality in the Western population and represents the most common cause for hospitalization in patients > 65 years old (Rydén-Bergsten and Andersson 1999). Since pulmonary congestion is a main reason for hospital admission in HF patients (Nieminen et al. 2006), diagnostic assessment of lung edema is of particular importance regarding disease monitoring, risk stratification and treatment control (Price 1975; Platz et al. 2016, 2017; Miglioranza et al. 2017). LUS has recently emerged as a powerful diagnostic tool in this patient population, as the accuracy of LUS to detect acute decompensated HF is markedly superior to the standard method of chest radiography, which notoriously suffers from low sensitivity (Maw et al. 2019). Consequently, the value of LUS in HF management has recently been highlighted in a position statement of the Heart Failure Association (Čelutkienė et al. 2018) and current HF guidelines (class IIb recommendation) (Ponikowski et al. 2016).

#### LUS in experimental cardiogenic edema

Despite the growing body of evidence demonstrating the usefulness of LUS in heart failure patients, its application in animal models has thus far been scarce. Although LUS has been successfully applied in dogs (Rademacher et al. 2014; Vezzosi et al. 2017) and pigs (Gargani et al. 2007; Jambrik et al. 2010), it has not been utilized in the evaluation of corresponding large animal models of cardiovascular disease. To date, the only study reporting the use of LUS in a preclinical model of heart failure has been in mice (Villalba-Orero et al. 2017). Clinically, HF is associated with characteristic clinical symptoms and signs like breathlessness or reduced exercise capacity, which are prerequisites for its diagnosis (Ponikowski et al. 2016). The difficulty of assessing such clinical characteristics in animal models is a major limitation of preclinical studies that generally limit themselves to providing evidence for cardiac dysfunction rather than for HF symptoms and signs. The study by Villalba-Orero and coauthors is as such a major advancement in reverse translation, as it highlights the potential usefulness of LUS for the detection of cardiogenic edema as the classic pulmonary manifestation of HF. In two mouse models of cardiovascular dysfunction—one of systolic dysfunction due to dilated cardiomyopathy and a second one of diastolic dysfunction as a result of diabetic cardiomyopathy—the authors demonstrated the usefulness of LUS to predict the onset of HF and to test treatment efficacy (Villalba-Orero et al. 2017). To this end, the authors established a score (“mouse LUS score”) that includes evaluation of sliding, profile, echo color, Z-lines, pleural thickness, pleural defects and pleural effusion, and correlated with both lung water content and cardiac function parameters (Villalba-Orero et al. 2017).

In patients, the most common cause for acute cardiogenic lung edema is MI. As LUS facilitates evaluation of pulmonary congestion and cardiogenic edema, it represents a powerful tool not only to rule out cardiogenic edema in prehospital emergency scenarios (Laursen et al. 2016) but putatively also for longitudinal monitoring of disease status, progression, resolution and therapeutic effectiveness in these patients. Yet, although animal models of MI, e.g., due to ligation of the left anterior descending coronary artery (Neye et al. 2012) are a mainstay of preclinical models of cardiovascular disease and are characteristically associated with the formation of cardiogenic lung edema as evident from increased wet-to-dry lung weight ratio at necropsy (Yin et al. 2008; Solymosi et al. 2013), LUS has so far not been applied to preclinical models of MI.

## Limitations

While first studies highlight the potential of LUS in preclinical studies, some word of caution is warranted as notable limitations apply with regard to technical problems and a lack of sufficient data regarding its application in small animals. First, due to the small area assessed per scan, pulmonary lesions may be missed. Although the relative proportion of the lung assessed may be greater in small animals than in humans, global evaluation still requires application of other techniques. Furthermore, application of X-ray or tomographic techniques often leads to unexpected findings (e.g., incidentalomas) that warrant further evaluation. In contrast, it appears unlikely that LUS will contribute substantially to the unexpected detection of pulmonary pathologies.

As animal models imitate specific pathologies, preclinical LUS is commonly used to confirm and quantify rather than to diagnose lung congestion or consolidation. This approach differs from LUS’ clinical application and does not allow for conclusions on the diagnostic ability of LUS. Moreover, a current major limitation of LUS in animal models is the significant lack of data. Although pilot studies demonstrate its feasibility in large and small animals, a systematic evaluation of LUS—especially in direct comparison with gold standard methods—is missing as of yet. As a consequence, the utility of LUS has only been documented in a couple of animal models. It remains to be shown whether the ultrasonic characteristics of distinct pathologies in humans are comparable with small animal models. As such, future studies utilizing a variety of preclinical models of pulmonary disease, standard operating procedures for small animal research and specific guidelines for the interpretation of LUS findings in rodents are required and need to be developed.

## Conclusion and perspectives

Over the past decade, LUS has surpassed and frequently replaced conventional chest radiography and even CT scans as a diagnostic tool for acute respiratory failure in intensive care medicine. LUS offers significant benefits over conventional imaging techniques in that it is rapid, non-invasive and radiation free. In contrast to CT scans and even chest X-ray, LUS is available in most hospitals around the world including developing countries and, as such, has been implemented as standard procedure for the diagnosis of ARDS according to the Kigali modification of the Berlin definition (Riviello et al. 2016). Due to its high mobility, LUS can be applied at the bedside, intraoperatively, or even in emergency medicine settings (Laursen et al. 2016). Most importantly, however, LUS generally outperforms conventional imaging techniques in terms of accuracy (i.e., sensitivity and specificity) and interobserver variability.

While these features have gained LUS a rapidly increasing appreciation and diagnostic relevance in clinical medicine, its application in preclinical animal models of pulmonary and cardiovascular disease is still in its infancy. This is both astonishing and regrettable at the same time—astonishing, as ultrasound has been introduced with great success into the preclinical arena for monitoring of cardiovascular disease and is by now a mainstay of any cardiovascular laboratory with a strong preclinical focus. Regrettable, as LUS, is one of a few non-invasive techniques in preclinical respiratory research (other than plethysmography and oxygen saturation measurements) and the only one that can reliably, sensitively, and semi-quantitatively detect pulmonary edema. The unique opportunity to study pulmonary edema formation and resolution longitudinally within a single animal poses a significant advantage over existing end-point parameters such as wet-to-dry lung weight ratio or lung histology. Importantly, longitudinal studies not only bear significant analytical advantages due to the possibility of intra- instead of interindividual statistical comparison but drastically reduce the number of animals required for time course analyses, thus serving one of the pillars of Russell and Burch’s 3R principle (replacement, reduction, refinement). In cases of cardiovascular disease and cardiogenic lung edema, LUS can simply be added to existing ultrasound routines, as echocardiography is already the most frequently applied technique for the functional evaluation of cardiovascular status and standard echocardiographic devices and probes for small animals are equally applicable for LUS. As such, assessment of pulmonary congestion and edema formation may be included into the routine sonographic workup without the need for additional animal handling.

In addition to cardiogenic lung edema, specific preclinical disease models or scientific questions where LUS may prove beneficial include (with no claim to be exhaustive), e.g., studies of alveolar fluid absorption, of lung atelectasis, or translational studies of ARDS in small animal intensive care units. First, quantitative analysis of impaired alveolar fluid absorption, a hallmark of ARDS, is presently largely restricted to isolated perfused human or animal lungs. Yet for reasons that are poorly understood, kinetics of alveolar fluid absorption differs considerably between in situ and in vivo conditions by a factor of approximately 4:1 (Fukuda et al. 2000). LUS bears the potential to monitor the process of alveolar fluid absorption in vivo in real time as decrease in the number of B-lines and/or ultimately as transition to A-lines in a non-invasive manner, thus yielding an exact time course in a physiologically relevant in vivo scenario. Second, current concepts for lung recruitment in mechanical ventilation are largely based on CT scans or—in preclinical settings—intravital microscopic observations showing atelectasis and subsequent recruitment (typically by elevation of positive end-expiratory pressure) of distal airspaces. What remains poorly understood, however, is whether these consolidated areas reflect regions of complete anatomical airspace collapse, or rather of partial or complete fluid filling, which notably cannot be distinguished by CT scan or intravital microscopy (Hubmayr 2002; Grune et al. 2019b). LUS with its ability to differentiate between tissue and fluid does not only allow to differentiate between the two phenomena but due to its real-time image, acquisition may also detect kinetic phenomena such as cyclic opening-and-collapse in a dynamic and unprecedented fashion. Finally, the poor translation of promising therapeutic strategies from mice to patients in ARDS has recently highlighted the need for more clinically relevant disease models that appropriately reflect the time course, multiple organ failure and iatrogenic life support interventions in clinical ARDS (Uhlig and Kuebler 2018). This recognition has fueled the development of small animal ICUs for the study of ALI/ARDS (Reiss et al. 2011) that may not only better replicate human disease but also necessitate the development of new monitoring techniques such as LUS that allow for longitudinal observation as compared with the classic end-point measurements 2 h after induction of ALI.

First data in experimental animals ranging in size from pigs to mice provide proof-of-principle that the demonstrated advantages of LUS in the clinical setting can be directly translated to the preclinical scenario, in that LUS in experimental settings is feasible and allows for an accurate, non-invasive detection of pulmonary edema. While these pioneering studies highlight the promise of LUS for preclinical research, further development of the technique is required to make it broadly applicable as a routine measurement in cardiopulmonary labs around the world. To this end, scoring systems such as the mouse LUS score developed by Villalba-Orero and colleagues will have to be refined, optimized and standardized (Villalba-Orero et al. 2017). Ideally, scoring systems should be developed that are easily applicable in different labs, different diseases and different species allowing for direct comparison of data between models and groups. Automated scoring systems such as the computer-assisted gray-scale analysis by Corradi and coworkers may help to eliminate interobserver variability and further speed up image analysis and interpretation (Corradi et al. 2013). In echocardiographic imaging, automated scoring systems in small animals have proven particularly beneficial for investigators with yet limited training in ultrasound imaging (Grune et al. 2019a). Finally, standard operating procedures for LUS imaging and scoring need to be developed and rigorously tested in a multicenter trial for accuracy and intra- and interobserver variability.

LUS holds great promise and there can be little doubt that its successful implementation into the clinics will soon be reproduced by a similar surge in applications in animal research. At present, LUS in preclinical research is only at the beginning, but—as Plato says—the beginning is the most important part of any work (Plato n.d.).
